# Personal familiarity of music and its cerebral effect on subsequent speech processing

**DOI:** 10.1038/s41598-020-71855-5

**Published:** 2020-09-09

**Authors:** Maïté Castro, Fanny L’héritier, Jane Plailly, Anne-Lise Saive, Alexandra Corneyllie, Barbara Tillmann, Fabien Perrin

**Affiliations:** 1 Auditory Cognition and Psychoacoustics Team – Lyon Neuroscience Research Center (UCBL, CNRS UMR5292, INSERM U1028), Centre Hospitalier Le Vinatier - Bâtiment 462 – Neurocampus, 95 boulevard Pinel, 69675 Lyon, Bron France; 2 Olfactory Coding and Memory Team – Lyon Neuroscience Research Center (UCBL, CNRS UMR5292, INSERM U1028), Lyon, France

**Keywords:** Perception, Emotion

## Abstract

Despite the obvious personal relevance of some musical pieces, the cerebral mechanisms associated with listening to personally familiar music and its effects on subsequent brain functioning have not been specifically evaluated yet. We measured cerebral correlates with functional magnetic resonance imaging (fMRI) while composers listened to three types of musical excerpts varying in personal familiarity and self (familiar own/composition, familiar other/favorite or unfamiliar other/unknown music) followed by sequences of names of individuals also varying in personal familiarity and self (familiar own/own name, familiar other/close friend and unfamiliar other/unknown name). Listening to music with autobiographical contents (familiar own and/or other) recruited a fronto-parietal network including mainly the dorsolateral prefrontal cortex, the supramarginal/angular gyri and the precuneus. Additionally, while listening to familiar other music (favorite) was associated with the activation of reward and emotion networks (e.g. the striatum), familiar own music (compositions) engaged brain regions underpinning self-reference (e.g. the medial prefrontal cortex) and visuo-motor imagery. The present findings further suggested that familiar music with self-related reference (compositions) leads to an enhanced activation of the autobiographical network during subsequent familiar name processing (as compared to music without self-related reference); among these structures, the precuneus seems to play a central role in personally familiar processing.

## Introduction

Music conveys one of the most powerful and multifaceted experiences to humans. Throughout one’s life, we develop strong relationships with different pieces of music, which become emotionally charged and which represent specific personally familiar stimulations. Personal familiarity refers to stimuli for which an individual has been in direct, real and personal contact, be it related to other individuals, a place or an object^1^. The concept of *personal* familiarity may be distinguished from that of *general* familiarity, such as the familiarity of famous individuals or popular songs^2–4^. Personally familiar stimuli can evoke the retrieval of episodic autobiographical memory (i.e., personally relevant events acquired in a specific spatiotemporal and emotional context, such as situations when listening to one’s favorite music) and/or semantic autobiographical memory (i.e., general knowledge of personal facts, such as the titles of the music)^5^. Given that the processing of personally familiar stimuli engages the autobiographical memory system, self-reference and familiarity need to be disentangled^6,7^.

While it has been shown that listening to self-selected popular songs can be associated with activity in the autobiographical memory network^8,9^, no research has yet investigated the neural distinction between self and familiarity in music, that is the cerebral mechanisms specifically associated when listening to personally familiar music, with and without self-referential content (i.e., one’s own compositions and favorite songs, respectively). Investigating the neural correlates of personal familiarity of music is of particular importance as it has been recently shown that personally familiar music listening has beneficial effects on pathologic cerebral functioning. For example, patients with visual neglect show enhanced visual attention when the experimental tasks were performed while listening to their preferred music in comparison to unpreferred music^10^. Personally familiar music has also beneficial effects on cognitive processes of patients with disorders of consciousness. Indeed, the probability to observe a cerebral or a behavioral response to the patient’s first name is enhanced when this personally familiar stimulus followed the presentation of the patient’s favorite music^11,12^. These findings suggest that the personally familiar characteristics of music improve the impaired higher-order cognitive functions, notably those involved in the processing of personally familiar items. However, the mechanisms underlying this effect are not known yet and need still to be investigated.

The aim of the present fMRI study was twofold. First, we aimed at investigating the cerebral correlates of listening to personally familiar music, with or without self-reference, i.e., composed (familiar own) music or favorite (familiar other) music, as compared to unknown (unfamiliar other) music. Composers were selected as participants to allow the study of brain regions associated with the processing of music with personal familiarity and self-reference (i.e. compositions). Second, we aimed at investigating the effect of personally familiar musical context (with or without self-reference) on the following processing of personally familiar stimuli (with or without self-reference), here names (i.e., first and last names of individuals). Thus three types of names were also used: own name (familiar own), name of a close friend (familiar other) or of an unknown (unfamiliar other) person. To meet both objectives, the composers were exposed to a set of experimental blocks that each consisted of a musical 30 s-excerpt (of one of the three types of stimuli), followed by a 30 s-sequence of 9 names (each of the three types being represented). This design is close to that of the studies that observed beneficial effects of music on name discrimination in patient populations^11^. We hypothesized that the brain networks associated with listening to names (with personal familiarity and/or self-referential contents) would be partly the same as the brain networks activated by the musical context (with personal familiarity and/or self-referential contents), for example when the composition (own music) is presented as the preceding context, the brain networks associated with listening to the own name would be more similar than the brain networks associated with listening to the own music than after a different musical context.

## Material and methods

### Participants

Sixteen right-handed composers who were French native speakers participated in the study (4 females; mean age: 27.3 ± 4.74 years; mean ± standard deviation). They reported not having any hearing problems or history of neurological disease. They have been composing music for on average 10.34 ± 5.09 years, practiced music for 18.63 ± 5.67 years and have participated in at least one publicly available album (e.g., CD). Their compositions covered various musical styles, such as pop, rock, electro, folk, classical music and experimental music.

### Stimuli

Auditory stimuli were of two types (i.e.*,* music and names) and varied in personal familiarity and self (familiar own, familiar other and unfamiliar other).

#### Musical excerpts

Participants filled out a questionnaire to indicate their 5 favorite own compositions, which served as familiar own music (OM), and their 5 favorite musical pieces or songs, which served as familiar other music (FM). In addition, a list of unknown artist or band names, mixed with famous artist and band names, was presented to each participant. The responses allowed selecting unfamiliar artists and bands for the preparation of the unfamiliar other and unknown music (UM). These UM pieces were matched with the 5 OM and the 5 FM by respecting the musical style (e.g. pop, rock, classical music), the instrumentation (e.g. guitar, bass and drum for a rock band, string quartet for a classical piece), the presence or absence of voice (and if possible the language of the lyrics used in the song), and the dynamics (tempo). For each participant, two representative excerpts (the beginning and a section of the refrain) of each of the 15 musical pieces or songs were selected, leading to 10 OM, 10 FM and 10 UM stimuli (prepared using Audacity® software, https://audacity.sourceforge.net/). The mean duration of the musical excerpts was 30.83 s ± 1.14 s (respectively, 30.68 ± 1.16 s for OM, 30.94 ± 1.20 s for FM and 30.86 ± 1.04 s for UM).

#### Names

The familiar own name (ON) was the participant’s full name, i.e., the first name followed by the last name. The familiar other name (FN) was the full name of a close friend, who was neither part of the participant’s family (to avoid the same last name) nor part of the participant’s musical projects (to avoid close connections to OM). It was selected based on a questionnaire where participants indicated three full names of close friends ranked by order of closeness. For each participant, the unfamiliar other name (UN) was created based on his/her familiarity ratings of a random list of first names and last names. Participants were asked to select those that were very familiar and evoked somebody they know, and UN were created from the first names and last names with the lowest ratings. UN, FN and ON were matched for the mean number of syllables (UN: 2.00 ± 0.73 and 2.25 ± 0.44; FN: 2.00 ± 0.56 and 2.12 ± 0.45; ON: 2.00 ± 0.73 and 2.44 ± 0.81, respectively for the first names and last names).

The names were recorded (wav format, 32 bits, 44.1 kHz) using a handheld recorder, and uttered by 6 female voices and 6 male voices, all unknown to the participants, with a neutral tone. For each participant, there were 36 stimuli, i.e., the 3 names (ON, FN and UN) recorded by the 6 female voices and 6 male voices. The mean duration of the name stimuli was 1.29 ± 0.21 s (respectively, 1.31 ± 0.26 s for ON, 1.26 ± 0.20 s for FN and 1.29 ± 0.17 s for UN).

For the experimental task, thirteen pseudo-names (PN) were created. PN contained legal French phonotactic arrangements, could be pronounced, but do not exist (e.g., Crusnio Porbinge). PN were created and uttered by the same 12 voices as the names and the mean duration of the stimuli was 1.43 ± 0.18 s.

All stimuli were normalized with Matlab R2011b (The MathWorks, Inc., Natick, MA, USA) to be at the same sound level, both within and between music and names. An A-weighting, that roughly mimics the external and middle ear transfer functions, was applied. During the experiment, stimuli were presented and responses were recorded using Presentation® software (Neurobehavioral Systems, Inc., Berkeley, CA, USA).

### Task and procedure

**Figure 1:**
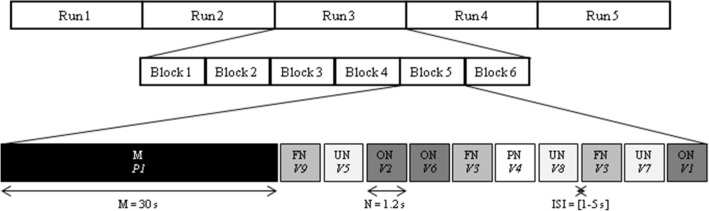
Experimental design. Schematic representation of the time course of the experimental session illustrated with one block. M: music condition, with OM, FM, or UM. P1: first part of a musical piece (P2: second part of a musical piece, not represented here). V1 to V9: 9 different voices played for the names (ON, FN and UN) and the pseudo-names (PN). *OM* familiar own music (composition); *FM* familiar other music (favorite); *UM* unfamiliar other music; *ON* familiar own names (participant’s own name); *FN* familiar other names (favorite person’s names), *UN* unfamiliar other names (names of unknown persons).

The experimental paradigm (Fig. 1) consisted of 5 runs of 6 blocks each. Each block consisted of one musical excerpt with a duration of 30 s, followed by a 30 s-sequence of 9 names (3 ON, 3 FN and 3 UN). Within a given block, the 9 names were presented in a pseudo-random way: one name could not be presented more than twice consecutively, and the 9 names were uttered by 9 different voices. The 9 names were presented in different orders between blocks and participants. For each run, the 6 blocks covered the 3 music conditions (2 OM, 2 FM and 2 UM), and the 2 excerpts of each musical piece were presented consecutively. The order of the 3 music conditions was counterbalanced between the 5 runs and between participants. The mean duration of the inter-stimulus-intervals (ISIs) between two names or between one music and one name or between one name and one music was 2.5 s, and the ISIs varied from 1 to 5 s, following an exponential distribution.

One or two PN were added to each sequence of names. To keep participants attentive, participants had to detect the PN within each name series, pressing the response button with their right hand as fast as possible. The PN were randomly presented between the second name and the ninth name, and if two PN appeared in the same block, they could not be consecutive.

Participants were instructed to listen carefully to the musical excerpts and the names presented via MRI-compatible headphones (MR confon Optime-1, Magdeburg, Germany), while keeping their eyes closed. One training block was presented to familiarize participants with the task (the same training block was played a second time if participants did not detect the PN) and to check the sound intensity. Then, the 5 runs were presented, lasting about 40 min, and the experiment ended with an 8 min anatomical MRI scan.

### Stimuli ratings and analyses

Immediately after the scanning session, participants re-listened (outside of the scanner) to the musical materials heard in the scanner. For each musical excerpt (presented in random order), participants made three judgments using 10-point scales: (1) pleasantness (from 1 = unpleasant, to 10 = pleasant), (2) familiarity (from 1 = unfamiliar, to 10 = familiar), and (3) the strength of music-evoked autobiographical memories (from 1 = absent, to 10 = numerous). For each of the three questions, a one-way ANOVA with personal familiarity (OM vs FM vs UM) as within-participant factor was performed, as well as LSD Fisher post-hoc tests when a significant effect was found.

Participants also listened to the 3 names (ON, FN, and UN) heard in the scanner, uttered by a different voice (not heard in the scanner), and had to judge the familiarity of these names with a 10-point scale (from 1 = unfamiliar, to 10 = familiar). A one-way ANOVA with personal familiarity (self-referential vs favorite vs unfamiliar) as within-participant factor, followed by LSD Fisher post-hoc tests, were conducted on familiarity judgments about names.

### fMRI scanning parameters

MRI images were acquired using a 1.5 T MAGNETOM Sonata whole-body imager (Siemens medical®, Erlangen, Germany). For functional imaging, we obtained 29 axial slices (all brain coverage, including the top of the cerebellum) using a T2*-weighted echo-planar sequence with the following parameters: resolution = 3.4 × 3.4 × 3.4 mm, time repetition (TR) = 2,500 ms, time echo (TE) = 50 ms, flip angle = 90°, field of view (FOV) = 220 × 220 mm. A high-resolution structural T1-weighted anatomical image was acquired with the following parameters: 176 sagittal slices, resolution = 1 × 1 × 1 mm, TR = 1970 ms, TE = 3.93 ms, FOV = 256 × 256 mm.

### fMRI data processing and analyses

The fMRI data were preprocessed and analyzed using SPM8 software (Statistical Parametric Mapping, https://www.fil.ion.ucl/spm/software/spm8, Wellcome Department of Cognitive Neurology, London, UK). The first 5 images of each functional run were discarded to allow for T2* equilibration effects. The remaining images were slice-time corrected, realigned to the acquired median volume, spatially normalized to the Montreal Neurological Institute (MNI) standard brain and smoothed with a 7 × 7 × 7-mm full width half maximum isotropic Gaussian kernel. Preprocessed data of each participant were analyzed with the standard general linear model (GLM) approach. The regressors for the musical excerpts (OM, FM, and UM) were modeled as epochs with a boxcar waveform convolved with the hemodynamic response function. The regressors for the names (ON, FN, and UN) were modeled as events with the canonical hemodynamic response function. The name events were defined based on their type (ON, FN, UN) and of the preceding musical excerpt (OM, FM, UM). The crossing of the three types of names with the three types of preceding music (3 × 3) led to the definition of nine types of regressors: participants’ own name (ON) following either own music (OM) or familiar other music (FM) or unfamiliar other music (UM), as well as familiar other names (FN) following the same three music conditions (that is, OM, FM, UN) or unfamiliar other names (UN) following the same three music conditions. The resulting nine regressors were labeled as follows (with “a” as abbreviation for “after the presentation of”): ONaOM, ONaFM, ONaUM, FNaOM, FNaFM, FNaUM, UNaOM, UNaFM and UNaUM.

First, the effect of the personal familiarity of the musical excerpts was investigated. The distinct activations for familiar own (self-referential) music and familiar other (favorite) music, respectively, were investigated with the contrasts (OM − UM) and (FM − UM). We also performed the conjunction [(OM − UM) ∩ (FM − UM)] in order to show the common activations for familiar (both own and other) music, in comparison with unfamiliar (other) music, which served as a control condition. The specificities of own versus other in familiar music perception were tested with the contrast (OM − FM). All the opposite contrasts, i.e. UM − OM, UM − FM and FM − OM, have been also conducted. Only significant results were presented with an uncorrected threshold of p < 0.001 and a minimal number of voxels of 20. We additionally reported all major clusters (k > 100) that survived the threshold of p < 0.05 corrected for multiple comparisons using the family-wise error (FWE) method, at the whole-brain cluster level.

Second, to study the influence of the personal familiarity of the musical excerpts on the processing of the names, several contrasts were performed. (1) We investigated the influence of the preceding musical context on *familiar own name* (ON) processing: (ONaOM − ONaUM) tested the effect of familiar own musical context (as compared to unfamiliar and other music), (ONaFM − ONaUM) tested the effect of familiar other musical context (as compared to unfamiliar music) and (ONaOM − ONaFM) tested the effect of familiar own as compared to familiar other music. (2) Similar contrasts were performed to investigate the influence of the preceding musical context on *familiar other name* (FN) processing: (FNaOM − FNaUM), (FNaFM − FNaUM), and (FNaOM − FNaFM). (3) The same three contrasts were performed to test the effect of the personal musical environment on subsequent *unfamiliar other name* (UN) processing: (UNaOM − UNaUM), (UNaFM − UNaUM) and (UNaOM − UNaFM). Only significant results were presented with an uncorrected threshold of p < 0.001 and a minimal number of voxels of 20.

### Ethics statement

All participants gave their written, informed consent and medical screening. The experiment was conducted in accordance with the guidelines of the Declaration of Helsinki and approved by the local ethics committee (CPP Sud-Est IV, n° 2012-A01209-34).

## Results

### Behavioral data

Participants detected correctly 99.46 ± 2.51% of the pseudo-names, and the mean percentage of false alarms (incorrect recognition of ON or FN or UN as PN) was 1.25 ± 5.09%. The mean response time of correct detection was 1.16 ± 0.68 s, remained stable across runs (F (4, 75) = 0.84; p = 0.50) and was not influenced by the preceding music conditions (F (2, 45) = 0.13; p = 0.88).

**Figure 2:**
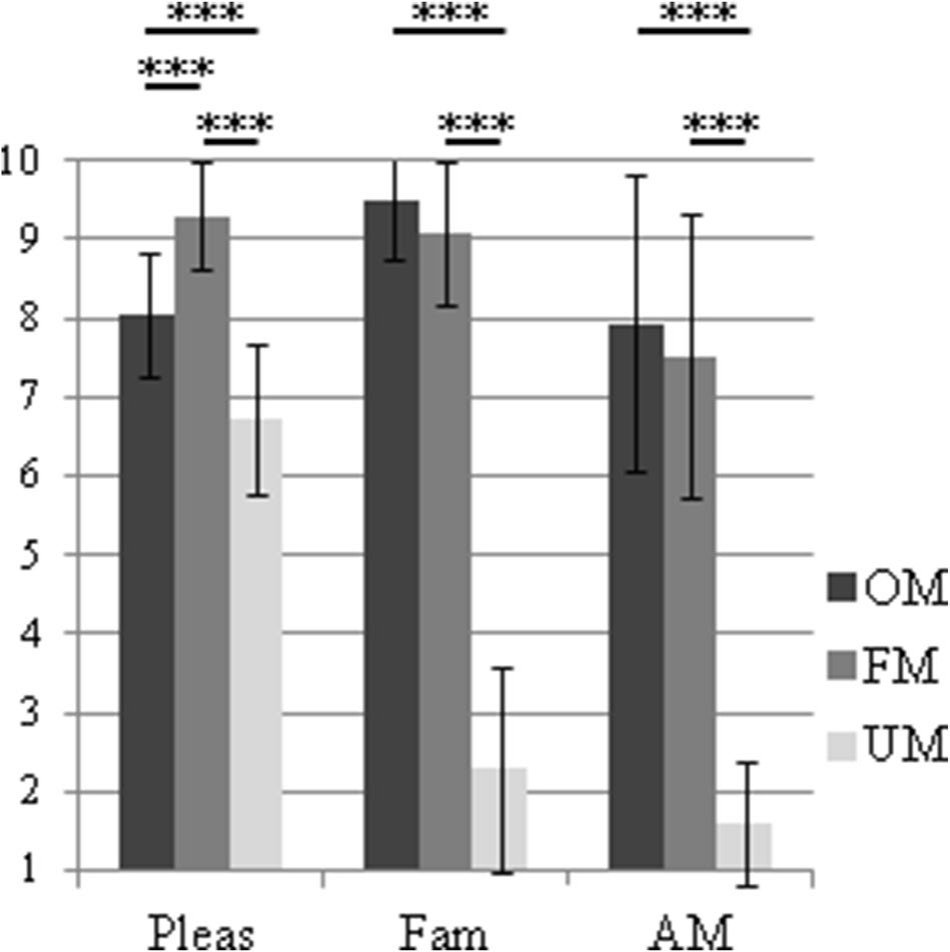
Average judgments ratings of the musical stimuli presented as a function of the three scales: pleasantness (Pleas), familiarity (Fam) and music-evoked autobiographical memories (AM). Error bars represent standard deviation of the mean. *OM* familiar own music (composition); *FM* familiar other music (favorite); *UM* unfamiliar other music.

For each of the three judgments’ scales, a significant effect of the personal familiarity of the musical excerpts was found for pleasantness, familiarity and music-evoked autobiographical memories ratings (F (2, 30) = 57.013; p < 0.001, F (2, 30) = 295.314; p < 0.001, F (2, 30) = 114.370; p < 0.001, respectively). The post-hoc Fisher LSD tests showed that (i) UM was rated as significantly less pleasant, less familiar and less autobiographical than OM and FM, respectively (ps < 0.0001), and that (ii) OM was rated as significantly less pleasant than FM (p < 0.0001). In addition, OM did not differ from FM for familiarity (p = 0.238) and autobiographical memory (p = 0.386). See Fig. 2: Pleasantness ratings: OM = 8.04 ± 0.78, FM = 9. 29 ± 0.67, UM = 6.72 ± 0.93; familiarity ratings: OM = 9.48 ± 0.73, FM = 9.08 ± 0.90, UM = 2.29 ± 1.31 and autobiographical memories ratings: OM = 7.94 ± 1.88, FM = 7.53 ± 1.79, UM = 1.59.

Participants rated the unfamiliar name as clearly less familiar (1.75 ± 2.27) than the highly familiar own name and the favorite name (10.00 ± 0.00 and 9.81 ± 0.54 respectively; ps < 0.0001).

### Neuroimaging data

**Figure 3:**
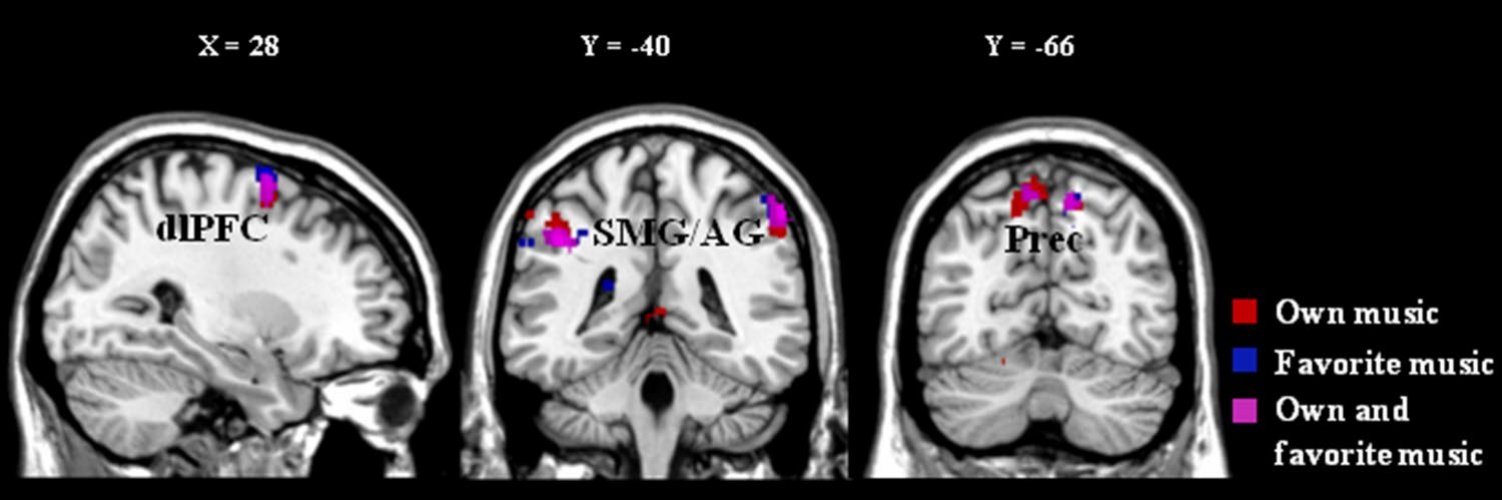
Brain activations related to the processing of familiar own music (composition), familiar other music (favorite) and brain activations common to both processing. Cluster of activations are in red for (OM − UM), in blue for (FM − UM) and in purple for [(OM − UM) ∩ (FM − UM)]. *OM* familiar own music (composition); *FM* familiar other music (favorite); *UM* unfamiliar other music; *dlPFC* dorsolateral prefrontal cortex; *SMG/AG* supramarginal gyrus/angular gyrus; *Prec* precuneus. The activations are superimposed on sections of a brain anatomical template (Colin 27 Average Brain, Stereotaxic Registration Model, Copyright (C) 1993–2009 Louis Collins, McConnell Brain Imaging Centre, Montreal Neurological Institute, McGill University).

Brain activations related to personal familiarity of music were presented in Table 1 and Fig. 3. Listening to music with personal familiarity, i.e., own and/or other familiar music ([(OM − UM) ∩ (FM − UM)], (OM − UM) and (FM –UM)) was associated with increased BOLD signals in the bilateral precuneus, the right dorsolateral prefrontal cortex, the bilateral supramarginal/angular gyri. Both own and other familiar music ((OM − UM) ∩ (FM − UM)) also activated the right anterior cingulate cortex and the right inferior frontal gyrus. The familiar own music (OM − UM) also induced activity changes in the right retrosplenial cortex, the left inferior frontal gyrus and the left motor regions, such as the supplementary motor area, the precentral gyrus and the cerebellum. The familiar other music (FM –UM) also activated the right retrosplenial cortex and the ventral striatum (including notably the nucleus accumbens).

**Table 1:** Brain activations related to the processing of familiar own music (composition) and familiar other music (favorite).

Regions	Laterality	x	y	z	Cluster size	Z
**[(OM − UM) ∩ (FM − UM)]**						
Precuneus	Bilateral	− 8	− 66	60	114	3.58
		12	− 64	56	105	3.83
Dorsolateral prefrontal cortex	Right	28	4	64	103	3.70
Supramarginal gyrus/Angular gyrus	Bilateral	− 44	− 40	38	280	4.26
		54	− 36	48	137	3.68**
Inferior Frontal gyrus (opercular part)	Right	46	8	2	33	3.62
Anterior Cingulate cortex	Right	2	26	24	27	3.55
**(OM − UM)**						
Precuneus	Bilateral	− 14	− 60	52	320	3.75**
		14	− 62	52	99	3.83
Dorsolateral prefrontal cortex	Right	26	2	56	27	3.83
Supramarginal gyrus/Angular gyrus	Bilateral	− 58	− 34	52	409	4.16**
		54	− 36	44	150	3.97
Inferior Frontal gyrus (opercular part)	Left	− 58	10	18	55*	3.26
Retrosplenial cortex	Right	4	− 42	6	21	3.65
Supplementary Motor Area	Left	− 4	− 4	62	75	3.59
Precentral gyrus	Left	− 60	4	26	55*	3.48
Cerebellum Lobule 6	Left	− 20	− 62	− 18	21	3.48
**(FM − UM)**						
Precuneus	Right	12	− 62	46	151	4.39**
Dorsolateral prefrontal cortex	Right	28	2	66	141	4.21**
Supramarginal gyrus/Angular gyrus	Bilateral	− 56	− 32	34	259	3.96**
		60	− 40	52	164	4.08**
Inferior Frontal gyrus (opercular part)	Right	50	8	4	66	3.96
Retrosplenial cortex	Right	12	− 34	26	39	4.06
Ventral striatum	Bilateral	12	− 10	− 4	20	3.82
		− 4	− 6	− 10	20	3.40

*FM* familiar other music (favorite); *OM* familiar own music (composition); *UM* unfamiliar other music.

*Number of voxels for the same cluster that regrouped different brain areas.

** Cluster level p value < 0.001 corrected.

**Figure 4:**
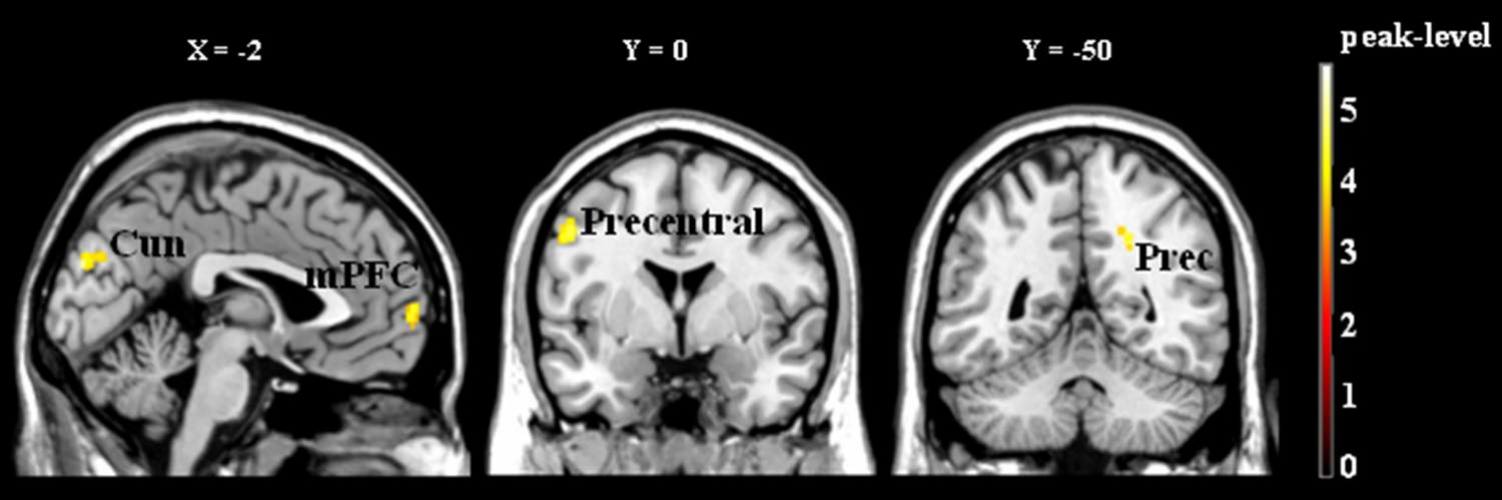
Brain activations related to the processing of familiar own music (composition) as compared to familiar other (favorite) music (OM − FM). Three main clusters are present: mPFC and visuo-motor regions (Cun and Precentral). *OM* familiar own music (composition); *FM* familiar other music (favorite); *Cun* cuneus; *mPFC* medial prefrontal cortex; *Prec* precuneus; *Precentral* precentral gyrus. The activations are superimposed on sections of a brain anatomical template (Colin 27 Average Brain, Stereotaxic Registration Model, Copyright (C) 1993–2009 Louis Collins, McConnell Brain Imaging Centre, Montreal Neurological Institute, McGill University).

Brain activations that distinguished own music from other music (both familiar) were presented in Table 2 and Fig. 4. As compared to familiar other music, familiar own music (OM − FM) activated the bilateral medial prefrontal cortex, but also the right precuneus, the left inferior temporal gyrus and near insular cortex, and the left visuo-motor areas (precentral gyrus and cuneus). No significant activation was observed for the inverse contrasts (UM − OM, UM − FM and FM − OM).

**Table 2:** Brain activations specific to the processing of familiar own music (composition) as compared to familiar other music (favorite).

Regions	Laterality	x	Y	z	Cluster size	Z
**OM − FM**						
Precuneus	Right	22	− 50	36	22	3.91
Medial prefrontal cortex	Bilateral	− 6	64	6	127	3.74
		10	64	18	28	3.84
Precentral gyrus	Left	− 52	0	40	52	3.77
Inferior Temporal gyrus	Left	− 48	− 42	− 14	23	3.55
Cuneus	Left	− 2	− 88	28	64	3.93
near Insular cortex	Left	− 32	− 26	28	72	3.62

*FM* familiar other music (favorite); *OM* familiar own music (composition).

**Figure 5:**
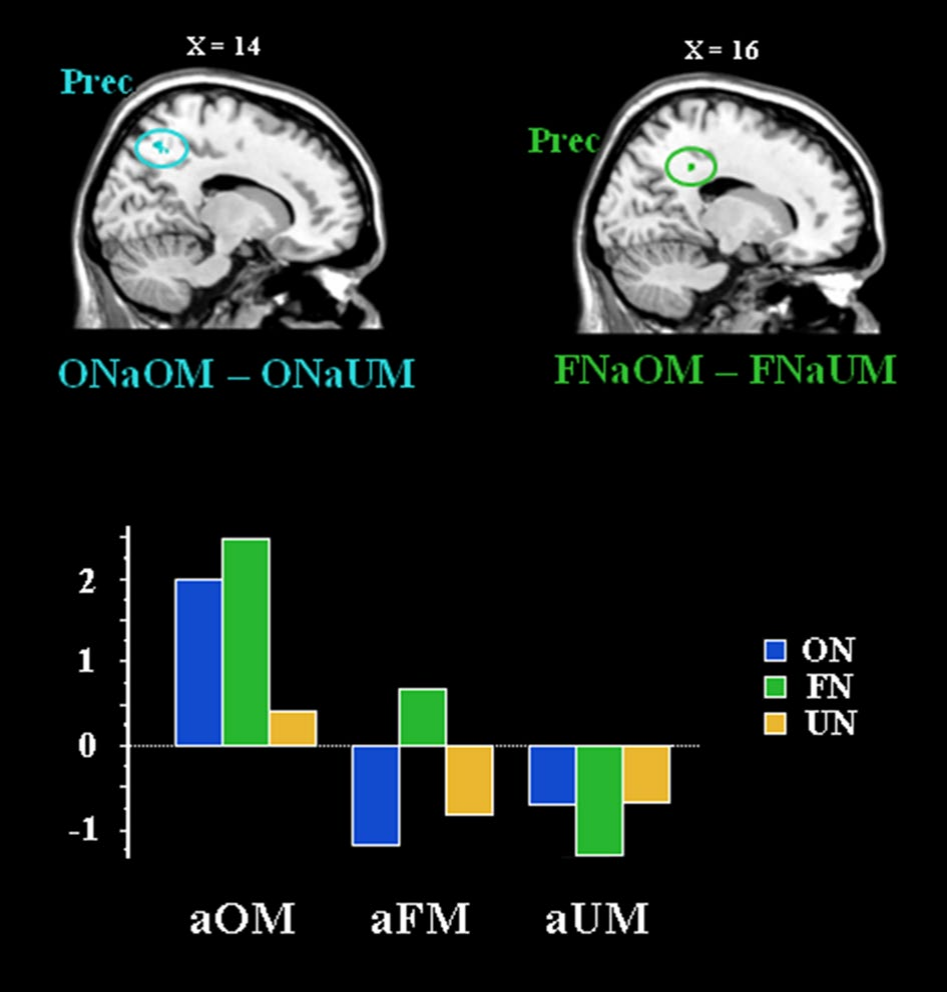
Brain activations related to the processing of familiar own name (participant’s own name) following familiar own music (composition), as compared to unfamiliar other music (ONaOM − ONaUM) (at the top, left) and the processing of familiar other name following familiar own music, as compared to unfamiliar music (FNaOM − FNaUM) (at the top, right). Parameters estimates (means) for the different names x musical contexts in the precuneus ROI (at the bottom). For a representation of the brain activations related to the processing of familiar own music as compared to unfamiliar other music (OM − UM), see clusters of activations in red in Fig. 3. *aOM* after familiar own music (composition); *aFM* after familiar other music (favorite); *aUM* after unfamiliar other music; *ON*, familiar own names (participant’s own name); *FN* familiar other names (favorite person’s names), *UN* unfamiliar other names (names of unknown persons); *Prec* precuneus. The activations are superimposed on sections of a brain anatomical template (Colin 27 Average Brain, Stereotaxic Registration Model, Copyright (C) 1993–2009 Louis Collins, McConnell Brain Imaging Centre, Montreal Neurological Institute, McGill University).

Brain activations related to the influence of the musical context on the processing of own names were presented in Table 3 and Fig. 5. The processing of the own name after having listened to familiar own music, as compared to the processing of the same stimulus after having listened to unfamiliar other music (ONaOM − ONaUM), activated the right precuneus, the left supramarginal/angular gyri, the left lingual gyrus and the left cerebellum. The processing of the own name after having listened to own (familiar) music, as compared to the processing of the same stimulus after having listened to other (familiar) music (ONaOM − ONaFM), engaged the left precuneus as well as the posterior cingulate cortex. No significant activation was obtained for the own name’s processing when it was preceded by familiar other music in comparison with unfamiliar other music (ONaFM − ONaUM).

**Table 3:** Brain activations related to the influence of the musical context on the processing of familiar own names (participant’s own name).

Regions	Laterality	x	y	Z	Cluster size	Z
**(ONaOM − ONaUM)**						
Precuneus	Right	14	− 60	48	23	3.78
Supramarginal gyrus/Angular gyrus	Left	− 30	− 36	40	38	3.80
Lingual gyrus	Left	− 22	− 74	− 8	29	3.75
Cerebellum Lobule 6	Left	− 20	− 62	− 18	27	3.97
**(ONaOM − ONaFM)**						
Precuneus	Left	− 12	− 40	64	28	3.92
Posterior Cingulate cortex	Right	22	− 32	46	56	4.53
Near Precuneus/Cuneus	Right	20	− 58	42	23	3.55

*ONaFM* familiar own name after the presentation of familiar other music (favorite); *ONaOM* familiar own name after the presentation of familiar own music (composition); *ONaUM* familiar own name after the presentation of unfamiliar other music.

Brain activations related to the influence of the musical context on the processing of familiar other names were presented in Table 4 and Fig. 5. The processing of the familiar other person’s name, after having listened to familiar own music, as compared to unfamiliar other music (FNaOM − FNaUM), was related to activations in the right precuneus, the right anterior cingulate cortex, the left hippocampus and sensorimotor regions: the left supplementary motor area, the left postcentral gyrus and the left putamen. The processing of familiar other names after having listened to own (familiar) music, in comparison with other (familiar) music (FNaOM − FNaFM), was associated with activations in the left precuneus and in sensorimotor regions: the left precentral and postcentral gyri, and the left cuneus. The processing of familiar other names after having listened to familiar (other) music, as compared to unfamiliar (other) music (FNaFM − FNaUM), activated the left anterior cingulate cortex and the right putamen.

**Table 4:** Brain activations related to the influence of the musical context on the processing of the familiar other names (favorite person’s names).

Regions	Laterality	x	y	Z	Cluster	Z
**(FNaOM** − **FNaUM)**						
Precuneus	Right	18	− 44	36	43	3.76
Anterior Cingulate cortex	Right	6	24	24	31	3.35
Postcentral gyrus	Left	− 50	− 20	24	55	3.62
Supplementary Motor Area	Left	− 4	− 20	56	36	4.17
Hippocampus	Left	− 30	− 22	− 16	24	4.13
Putamen	Left	− 30	0	8	24	3.84
**(FNaOM** − **FNaFM)**						
Precuneus	Left	− 12	− 42	70	43	3.89
Postcentral/Precentral gyri	Left	− 34	− 22	50	53	4.67
Cuneus	Left	− 12	− 70	22	55	3.83
**(FNaFM** − **FNaUM)**						
Anterior Cingulate cortex	Left	− 12	6	34	33	3.93
Putamen	Right	28	2	6	45	4.36

*FNaFM* familiar other name (favorite) after the presentation of familiar other music (favorite); *FNaOM* familiar other name (favorite) after the presentation of familiar own music (composition); *FNaUM* familiar other name (favorite) after the presentation of unfamiliar other music.

Investigation of the processing of the unfamiliar other name, according to the preceding personal musical context, did not reveal any significant activation, except for the contrast (UNaFM − UnaUM) for which one cluster situated in the midbrain was observed.

## Discussion

The present study aimed at investigating the neural correlates of the personal familiarity and self of musical material, as well as the effect of personal familiarity and self of music on the cerebral correlates associated with subsequent processing of names with personal familiarity and/or self-reference. To allow for defining the three types of music, notably the familiar own music, we selected individuals who create their own music, that is, composers. As composers are specific experts in music, we cannot rule out the hypothesis that the results are not entirely generalizable to a population of nonmusicians. However, while functional and structural brain differences have been observed between musicians and nonmusicians^13,14^, the here observed result patterns were in agreement with other findings related to neural correlates of self-processing and autobiographical memory^1^, not necessarily the target of musical expertise specifically.

First, we observed that personally familiar (own and/or other) music listening was associated with the activation of the precuneus, the dorsolateral prefrontal cortex and the supramarginal/angular gyri. Second, we showed that the brain correlates of the processing of personally familiar names (with or without self-reference) differed as a function of the personal familiarity and/or self-content of the preceding musical contexts.

### Brain activations associated with listening to personally familiar music

Some neuroimaging studies have shown that *familiar* (vs unfamiliar) music has been associated with the activation of a large fronto-temporo-parietal network^8,15–17^. This is in agreement with our present findings: listening to familiar (own and/or other) music, which were both judged as being more familiar than unfamiliar music, induced activations mainly in the supramarginal gyrus/angular gyri, the precuneus and the dorsolateral prefrontal cortex. The two latter brain structures have been previously involved in vivid *autobiographical memory retrieval* (for a review, see^18^), in link with our behavioral data showing that familiar (own and other) music obtained higher autobiographical scores than unfamiliar music. This suggests that listening to music with personal familiarity (composition and/or favorite music) is probably linked to the retrieval of contexts during which the music was composed or previously encountered.

Weak activations (i.e., only significant at the uncorrected threshold level, p < 0.001) were also in agreement with other studies investigating music processing. For example, favorite (familiar other) music activated the *reward* network (here the ventral striatum), as previously reported for music with emotion^19–23^. Likewise, listening to own vs other (familiar) music is mainly associated with activation in the bilateral medial prefrontal cortex, a region of the brain that supports *self-referential* processes^1,6,24–33^, and with various *visuo*-*motor* regions (in the supplementary motor area, the cerebellum, the precentral gyrus and cuneus), probably related to composers knowing the musical excerpts from a procedural point of view. This is consistent with the study of Meister and collaborators^34^ who showed activation of motor and visual areas when musicians were engaged in a task of music imagery.

### Effect of personally familiar music on subsequent personally familiar name processing

Numerous research has demonstrated beneficial effects of music listening on multiple brain functions, both for normal^35^ and pathologic cerebral functioning^36^. The beneficial effects of music could be explained by the fact that music processing involves distributed cortical systems, with some neural resources being shared with other sensory, cognitive or motor functions^37,38^. While some studies have proposed that music has a general effect on arousal and mood^39–41^, other studies have shown that the intrinsic characteristics of music may be responsible for the specific beneficial effects. For example, rhythm increases the recovery of gait and arm movements in hemiparetic stroke patients or Parkinson’s disease^23,42^, and melodic intonation (including its rhythmic component) improves spontaneous speech output, articulation, and naming in aphasic patients^43,44^. Personal relevance embedded in music seems also to create some of the beneficial effects on brain functioning. As previously shown for patients with disorders of consciousness^11,12^, listening to personally familiar music has an effect on the subsequent processing of personally familiar names.

While some of the results obtained in the present study for name processing should be interpreted with caution (as they did not pass a standard corrected threshold level), they suggest that the cerebral mechanisms associated to name processing are influenced by the type of preceding musical context. Indeed, personally familiar name (own and favorite) processing was associated to enhanced activations in structures that were previously activated for familiar own music listening. This was the case for the precuneus, the supramarginal/angular gyri and the cerebellum for own music processing and for own name processing following own music listening, as well as for the precuneus and the supplementary motor area for own music processing and favorite name processing following own music. Thus, the present study suggests that the cerebral structures activated during personally familiar and own music listening, notably the autobiographical network and the sensorimotor regions, might be reactivated afterwards during the processing of personally familiar names. This finding further suggests that after having listened to personally familiar music, the perception of personally familiar names is associated with extended cognitive processes linked to musical familiarity, imagery, self or autobiographical retrieval.

### Precuneus: a central role for the processing of personally familiar stimuli

In the present study, the precuneus was activated in most comparisons between personally familiar and unfamiliar stimuli: during familiar (own and/or other) music listening, but also for familiar (own and other) name processing following own music listening. These findings suggest that the precuneus is very often associated with the processing of personally familiar stimuli (in music or language), independently of the presence or absence of direct self-related contents. This is reminiscent of the neural distinction that has been suggested between anterior and posterior cortical midline regions for self and personal familiarity, respectively^7^. Our present study also suggests that the enhanced activation of the precuneus in personally familiar name processing is conditioned by its previous activation during personally familiar music listening.

The precuneus is a multimodal associative area sharing connections with subcortical regions^45^ and is often seen as a hub between parietal and prefrontal regions^46–48^. Previous functional neuroimaging studies have demonstrated the core role of the precuneus in the default mode network^49,50^ and in several highly integrated functions, including mental imagery, autobiographical memory retrieval and self-related processing^45,51^. For example, the precuneus was activated during the perception of personal familiar stimuli with or without self-reference (e.g., the own first name^31^ or familiar voices^52^) and is deactivated during loss of consciousness^53–55^.

These previous studies and the present study suggest a central role of the precuneus in personally familiar component integration. The present study also suggests that its activation is enhanced during the processing of personally familiar names when a personally familiar music has been previously presented. This could explain the beneficial effect of favorite music on own name perception that has been observed in patients with disorders of consciousness^11^.

## Data Availability

The results generated during the current study are available from the corresponding author on request.
